# Leadless vs. transvenous pacemakers following transcatheter aortic valve replacement: a dual-centre propensity score-matched observational study

**DOI:** 10.1093/europace/euaf264

**Published:** 2025-10-29

**Authors:** Sandrine Venier, Mouna Ben Kilani, Mailys Olivier, Grégoire LeGuily, Adrien Carabelli, Rémi Bénali, Antoine Desbiolles, Youssou Diouf, Peggy Jacon, Pascal Defaye

**Affiliations:** Department of Cardiology, Grenoble Alpes University Hospital, CS10217, Cedex 9, Grenoble 38043, France; University Grenoble Alpes, INSERM, CHU Grenoble Alpes, LRB, 38000, Grenoble, France; Department of Cardiology, Angers University Hospital, Angers, France; Department of Cardiology, Grenoble Alpes University Hospital, CS10217, Cedex 9, Grenoble 38043, France; Department of Cardiology, Angers University Hospital, Angers, France; Department of Cardiology, Grenoble Alpes University Hospital, CS10217, Cedex 9, Grenoble 38043, France; University Grenoble Alpes, INSERM, CHU Grenoble Alpes, LRB, 38000, Grenoble, France; Department of Cardiology, Grenoble Alpes University Hospital, CS10217, Cedex 9, Grenoble 38043, France; Department of Cardiology, Grenoble Alpes University Hospital, CS10217, Cedex 9, Grenoble 38043, France; Department of Cardiology, Grenoble Alpes University Hospital, CS10217, Cedex 9, Grenoble 38043, France; Department of Cardiology, Grenoble Alpes University Hospital, CS10217, Cedex 9, Grenoble 38043, France; Department of Cardiology, Grenoble Alpes University Hospital, CS10217, Cedex 9, Grenoble 38043, France; University Grenoble Alpes, INSERM, CHU Grenoble Alpes, LRB, 38000, Grenoble, France

**Keywords:** Transcatheter aortic valve replacement, Leadless pacemaker, Transvenous pacemaker

## Introduction

Transcatheter aortic valve replacement (TAVR) has significantly improved the management of severe aortic stenosis in elderly and high-risk surgical patients. However, conduction disturbances remain a frequent complication following TAVR, with up to one-third of patients requiring permanent pacemaker implantation.^1,2^ Leadless pacemakers (LPMs) have emerged as a promising alternative to traditional transvenous pacemakers (TPMs).^3^ Real-world data showed lower long-term complication rates with LPMs in high-risk subgroups,^4^ although Alhuarrat *et al*.^5^ reported higher in-hospital mortality and complications compared with transvenous systems. Yet, comparative data on the use of LPMs vs. TPMs in the post-TAVR setting remain limited. This dual-centre observational study aimed to evaluate procedural and clinical outcomes associated with LPM and TPM implantation early after TAVR.

## Methods

This retrospective analysis included consecutive patients who underwent PM implantation within a few days following TAVR at two French university hospitals (Grenoble and Angers) between May 2019 and May 2024. Only patients receiving a single- or dual-chamber TPM or a LPM were included. Patients requiring cardiac resynchronization therapy or implantable cardioverter-defibrillator for class I or IIa indications were excluded.^6,7^ All procedures were performed by senior electrophysiologists with more than 10 years of experience. Baseline clinical, electrocardiographic, and procedural data were collected. A 1:1 propensity score matching (PSM) was performed based on nine variables. The final matched cohort included 158 patients (79 per group). Matched cohorts were compared with respect to procedural metrics, hospital stay, complications, and long-term outcomes. The choice of LPM vs. TPM was guided by clinical context and team consensus.

This research has been registered in accordance with French regulations, meets the requirements of the CNIL reference methodology 004, and was declared to the local ethics committee.

The primary endpoint of the study was a composite of pacemaker-related complications, device-related reinterventions, and infections. A competing risk analysis was performed for this composite outcome, with all-cause mortality considered as competing event. Secondary endpoints included all-cause mortality, procedural metrics, and hospitalizations for heart failure.

## Results

Out of 200 eligible patients, 187 were included in the final analysis: 80 received an LPM and 107 a TPM. In the LPM cohort, 77 patients (96.3%) received a Micra-VR device and 3 (3.7%) a Micra-AV. In the TPM group, 25% received single-chamber and 75% dual-chamber devices.

**Figure 1:**
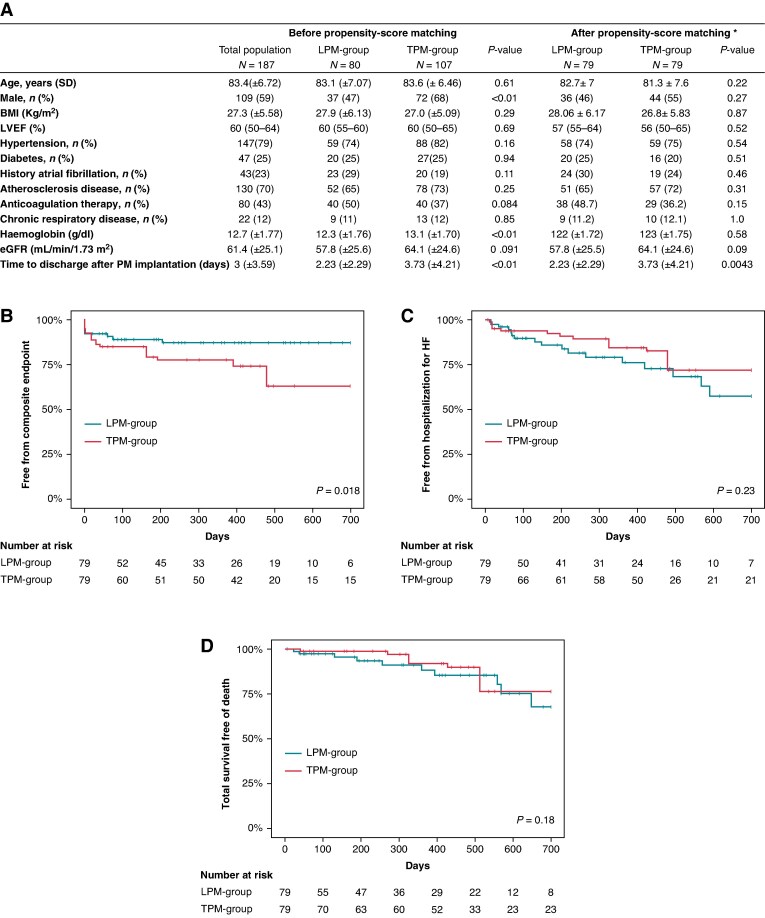
(*A*) Baseline characteristics. Categorical values are expressed as *n* (%). Continuous values are displayed as mean ± standard deviation or median [interquartile range]. BMI, body mass index; eGFR, estimated glomerular filtration rate; LBBB, left bundle branch block; RBBB, right bundle brunch block; AV, atrioventricular. *Age, sex, BMI, hypertension, diabetes, LVEF, history of AF, chronic pulmonary disease, and atherosclerosis were the variables used for PSM. (*B*) Composite endpoint of device-related complications, infections, and reinterventions. (*C*) Heart failure hospitalizations. (*D*) All-cause mortality, during follow-up.

*Figure 1A* summarizes the baseline characteristics. All patients had undergone TAVR with new-generation valves (*Edwards Sapien® 61.5%*, *Evolut® 36.4%*, and *Navitor® 2.1%*). Complete or high-degree atrioventricular (AV) block was the most frequent indication for pacing, observed in 136 patients (73%). The predominant indication for pacemaker implantation was complete or high-degree AV block, whereas less frequent indications included new-onset LBBB with PR prolongation or prolonged HV interval on electrophysiological study, second-degree AV block, and post-TAVR bradycardia. Median ventricular pacing percentage at 6 weeks was 20% in the LPM group and 27% in the TPM group. After PSM, 158 patients remained (79 per group).

### Procedure-related characteristics in propensity-matched cohorts

Procedure time, measured from room entry to exit, was significantly shorter in the LPM group (mean 60.9 ± 26.5 min) than in the TPM group (mean 94.0 ± 36.9 min, *P* < 0.001). A less fluoroscopy time was observed in the LPM group (2.23 ± 2.29 min vs. 3.73 ± 5.57 min, *P* = 0.01). Hospital stay post-implantation was also reduced in the LPM group (2.23 ± 2.29 vs. 3.73 ± 4.21 days, *P* = 0.0043).

### Short-term outcomes and follow-up in propensity-matched cohorts

In-hospital complication rates were not significantly different (5% LPM vs. 10% TPM, *P* = 0.37). In the TPM group, complications included two deep vein thrombosis, three pneumothoraces, and three lead dislodgements, whereas in the LPM group, they consisted of two arteriovenous fistulas and two cases of device dysfunction (one incomplete dislodgement and one case of unacceptably high pacing threshold identified on Day 1). During a median follow-up of 12 months [IQR: 3–18], the composite endpoint of device-related complications, infections, and reinterventions was significantly less frequent in the LPM group (HR 0.31; 95% CI 0.13–0.78; *P* = 0.018) (*Figure 1B*). The absolute number of patients experiencing the composite endpoint was 9 in the LPM group (11%) vs. 18 in the TPM group (23%). There were no significant differences in all-cause mortality (HR 0.579; 95% CI 0.23–1.43; *P* = 0.23) or heart failure hospitalization (HR 0.53; 95% CI 0.21–1.34; *P* = 0.18) (*Figure 1C* and *D*). Infections requiring rehospitalization after pacemaker implantation were more frequent with TPMs (10%) than with LPMs (2.5%), mostly non–device-related (bloodstream infections with suspected digestive or peripheral vascular origins), although two confirmed lead-related infections occurred in the TPM group. Device-related reinterventions were comparable between the two groups (3.8% in the LPM group vs. 3.7% in the TPM group; *P* = 1). A total of six events were reported: one atrial lead replacement, one LPM dysfunction managed by implantation of a dual-chamber device, one TPM removal due to infection, and three upgrades to cardiac resynchronization therapy (CRT).

## Discussion

Our study supports the safety and feasibility of LPMs in patients needing early pacing after TAVR. Despite the frailty of this population, LPMs were associated with faster recovery and a significantly lower rate of infection and device-related adverse events, without compromising survival or heart failure outcomes. These results align with recent observational studies showing reduced complication rates with LPMs in TAVR populations, such as Ueyama *et al*.^8^ while contrasting with administrative database analyses suggesting higher complication rates for LPMs in less-selected populations.

The elevated rate of heart failure hospitalization in our cohort is more likely attributable to the baseline risk profile of post-TAVR patients than to pacing, as evidenced by the low CRT upgrade rate and modest pacing burden. While dual-chamber pacing offers AV synchrony, its superiority in this elderly population remains unproven.^9,10^ In this frail population, the shorter hospital stay observed in LPM recipients is clinically relevant.

### Limitations

This was a retrospective observational study, and although PSM was performed to mitigate bias, residual confounding remains possible. It was not powered to detect differences in mortality or heart failure hospitalization. As such, results should be interpreted as hypothesis generating.

## Conclusion

In this dual-centre, propensity-matched observational study, LPM implantation following TAVR was associated with shorter procedure times, reduced hospital stays, and a lower incidence of the composite endpoint compared with TPM.

## Data Availability

The data underlying this article will be shared on reasonable request to the corresponding author.
